# Synthesis and Photocatalytic Decolorization Performance of a THPP/TiO_2_/Ag@ACF Aerogel Multiphase Composite

**DOI:** 10.3390/gels12100858

**Published:** 2026-09-22

**Authors:** Gang Wang, Yu Wang, Yi Yang, Zhihua Cheng, Rong Liu, Lina Chang, Guangyu Zhang, Jiamu Dai, Du Nie, Chuanfeng Zang

**Affiliations:** 1 School of Textile and Clothing, Nantong University, Nantong 226019, China; wanggang@stmail.ntu.edu.cn (G.W.); wangy@stmail.ntu.edu.cn (Y.W.); yangy@stmail.ntu.edu.cn (Y.Y.); chengzh@stmail.ntu.edu.cn (Z.C.); rong063@ntu.edu.cn (R.L.); zgyu85@ntu.edu.cn (G.Z.); flory2114@hotmail.com (D.N.); 2 Suzhou Fiber Inspection Institute Co., Ltd., Suzhou 215128, China; wytsmile0328@163.com; 3 Jiangsu Advanced Textile Engineering Technology Center, Nantong 226006, China

**Keywords:** activated carbon fiber aerogel, THPP/TiO_2_/Ag, photocatalytic decolorization, interfacial processes

## Abstract

Recoverable photocatalysts with broad optical response are of interest for practical water treatment. A 5,10,15,20-tetrakis(4-hydroxyphenyl)porphyrin (THPP)/TiO_2_/Ag composite was prepared hydrothermally and immobilized on activated carbon fiber (ACF) aerogel by silane-assisted dip coating. The design combined potential THPP photosensitization and Ag-mediated interfacial effects with TiO_2_ photocatalytic sites and an adsorptive, recoverable ACF support. Structural and spectroscopic characterization supported incorporation of the components and changes in optical response and emission behavior. Overall apparent methylene blue removal reached 88.7% under UV irradiation and 88.0% under xenon-lamp irradiation. The concentration profiles were approximately fitted using an apparent pseudo-first-order model. Increasing the number of immersion cycles did not further improve the photocatalytic performance. These results demonstrate adsorption-assisted dye removal using an immobilized multiphase composite. The proposed charge-transfer pathway is presented as a mechanistic interpretation rather than a directly established electronic configuration.

## 1. Introduction

Water pollution poses persistent risks to ecosystems and public health [1,2,3,4]. Activated carbon fibers (ACFs) are widely used as adsorbents for water and gas treatment and have also been investigated as supports for photocatalysts [5,6,7,8,9,10]. 5,10,15,20-Tetrakis(4-hydroxyphenyl)porphyrin (THPP) has a conjugated molecular structure that supports strong light absorption and photoinduced electron transfer [11,12]. However, the performance of THPP alone remains limited in many photocatalytic systems [13,14]. Titanium dioxide (TiO_2_) is widely used for environmental photocatalysis because of its chemical stability, activity, and relatively low cost [15,16,17,18,19]. Its performance depends on the generation, migration, and interfacial reaction of photogenerated charge carriers before recombination occurs [20,21,22].

Pristine TiO_2_ absorbs mainly ultraviolet light, uses only a small fraction of the solar spectrum, and often exhibits rapid electron–hole recombination and particle agglomeration [23,24,25,26]. Porphyrin sensitization and interfacial modification have therefore been explored to extend the spectral response and alter charge-transfer behavior. Feng et al. [27] incorporated Cu nanoparticles into a TiO_2_/THPP system and proposed a Type-II pathway for visible-light-driven hydrogen evolution. Jing et al. [28] coupled zinc tetracarboxyphenylporphyrin with THPP to regulate the interfacial electric field and reduce interfacial defects. Xi et al. [29] used coordination between THPP and Ni^2+^ on NiO to improve interfacial coupling. These studies demonstrate the potential of porphyrin–semiconductor interfaces, while also showing that the operative charge-transfer pathway depends on the specific composition and supporting evidence.

Silver nanoparticles can contribute antimicrobial activity and modify light absorption and interfacial electron transfer in TiO_2_-based photocatalysts [30,31]. Their localized surface plasmon resonance may increase visible-light absorption, while the metal–semiconductor interface can influence charge separation and transport [32,33,34,35,36]. However, the stability and mechanistic role of Ag depend on particle size, oxidation state, dispersion, and reaction conditions.

Porphyrin sensitization, metal modification, and carbon-based supports have been investigated in TiO_2_-based photocatalysts. The present work combines these components in an immobilized ACF aerogel architecture and compares samples prepared using one and two immersion cycles, with attention to both dark adsorption and irradiation-stage degradation.

From the perspective of practical water treatment, the physical form of a photocatalyst is also important. Dispersed photocatalytic powders provide a large accessible surface area but require subsequent separation from treated water, whereas immobilization on a porous fibrous support can simplify catalyst recovery and simultaneously provide adsorption sites for pollutant enrichment. ACF aerogels are particularly suitable as supports because their interconnected fibrous structure provides a large accessible interface, adsorption capability, and convenient macroscopic handling. Therefore, integrating a light-responsive photocatalytic phase with an adsorptive ACF framework provides an opportunity to combine pollutant enrichment, photocatalytic decolorization, and catalyst recovery within a single material. Nevertheless, the influence of the loading process and the interaction between adsorption and irradiation-induced removal in such porphyrin/TiO_2_/metal/ACF multiphase systems remain insufficiently clarified.

In this study, THPP and Ag were combined with TiO_2_ and immobilized on activated carbon fiber aerogel to obtain THPP/TiO_2_/Ag@ACFag (Figure 1). The design was intended to combine potential THPP photosensitization, TiO_2_ photocatalytic sites, Ag-mediated interfacial effects, and adsorption and physical recoverability provided by the ACF support [37,38,39,40].

**Figure 1:**
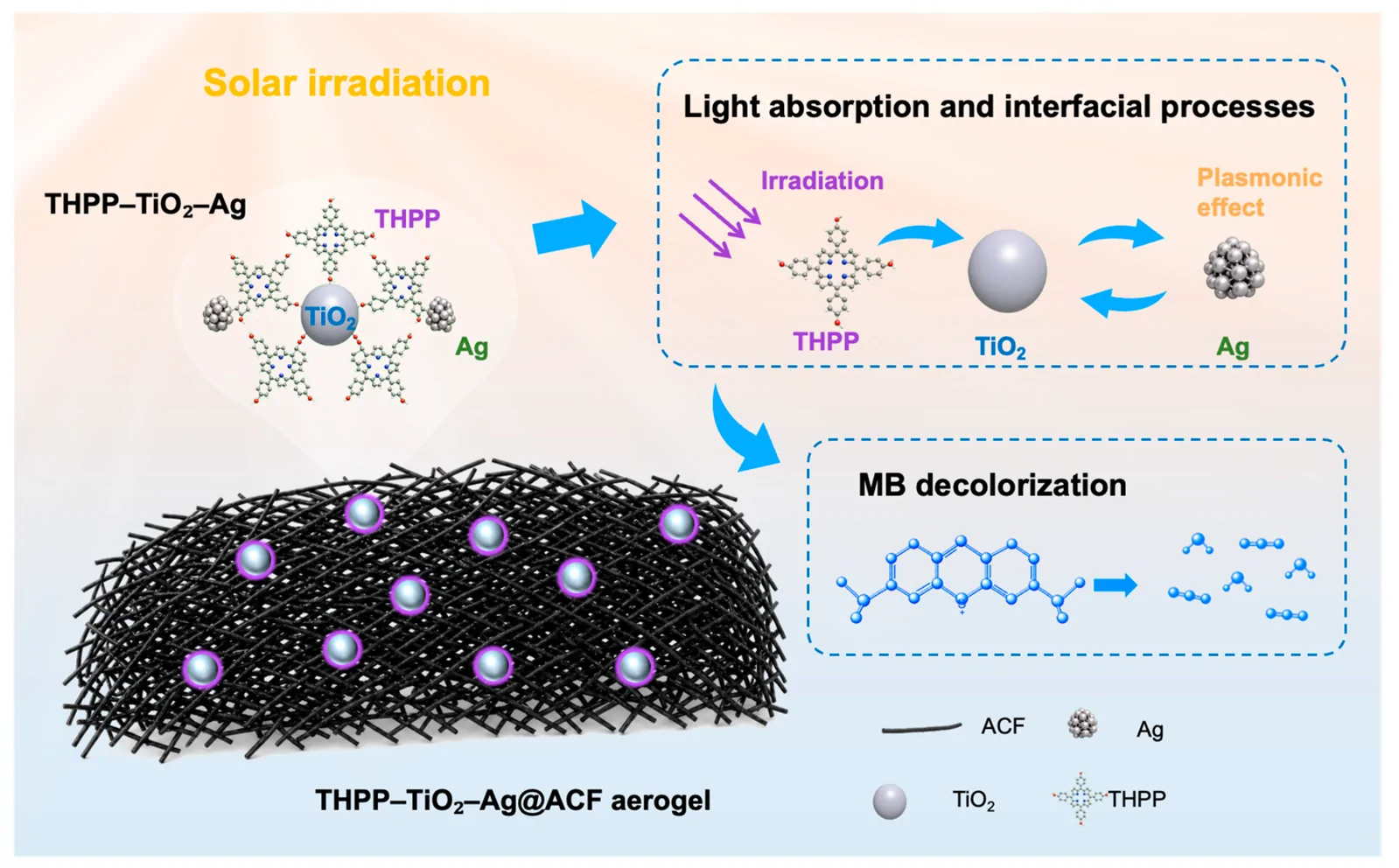
Proposed interfacial processes in the ACF aerogel-supported THPP/TiO_2_/Ag composite.

## 2. Results and Discussion

### 2.1. Morphology and Elemental Distribution

Figure 2 summarizes the synthesis and characterization of the THPP/TiO_2_/Ag composite. The precursor containing THPP, tetrabutyl titanate, and Ag nanoparticles was hydrothermally treated to obtain the composite powder (Figure 2a). SEM images (Figure 2b,c) show irregular aggregates composed of densely packed nanoparticles with rough surfaces. These observations establish the morphology of the powder but do not, by themselves, identify a specific heterojunction type.

TEM images (Figure 2d,e) show hierarchical aggregates assembled from nanoparticles and lattice fringes with reported spacings of 0.355, 0.354, and 0.359 nm, assigned to anatase TiO_2_ (101). The reported lattice continuity at the Ag/TiO_2_ interface is consistent with intimate crystalline contact, but lattice-fringe continuity alone does not establish an electronic junction or a direction of charge transfer [41,42,43]. EDS mapping results show Ti, *C*, N, and Ag across the analyzed region (Figure 2f–j), while the corresponding analysis indicates a relatively low Ag content (Figure 2k).

The FTIR spectra are shown in Figure 2l. Bands reported at 3444, 1637, and 624 cm^−1^ were assigned to surface O–H stretching, H–O–H bending of adsorbed water, and Ti–O–Ti vibrations, respectively. The THPP/TiO_2_/Ag spectrum also contained bands at 2919 and 2849 cm^−1^ and features at 1634, 1460, and 1036 cm^−1^, which were assigned to vibrations associated with the porphyrin framework [44]. Features near 650 and 460 cm^−1^ were attributed to Ti–O vibrations. The spectral differences are consistent with interaction or association between THPP and TiO_2_, although the bonding mode requires independent confirmation.

Figure 2m shows the reported XRD patterns of THPP/TiO_2_ and THPP/TiO_2_/Ag alongside separate reference patterns. Reflections at approximately 25.3°, 37.8°, 48.0°, 53.9°, 55.1°, 62.7°, 68.8° and 75.0° were assigned to anatase TiO_2_. The feature near 25.3° was assigned to the anatase (101) reflection. Peaks at 38.1°, 44.3°, 64.4°, and 77.4° were assigned to metallic Ag (PDF No. 65-2871). Compared with THPP/TiO_2_, the Ag-containing composite exhibited lower peak intensities and broader features while retaining the reported anatase reflections.

### 2.2. Morphology and Structure of THPP/TiO_2_/Ag@ACFag

Figure 3a illustrates silanization and dip coating of THPP/TiO_2_/Ag particles onto ACF aerogel. The SEM image in Figure 3b shows that the coated fiber surface is coated with particles, and the fibrous framework remains visible. This morphology is consistent with deposition of particulate material on the ACF aerogel surface.

The EDS maps in Figure 3c–g show signals assigned to C, Ti, O, N, and Ag in the analyzed region. Carbon corresponds primarily to the ACF aerogel substrate, while the Ti and O signals are consistent with TiO_2_. The N signal is consistent with the presence of THPP, and Ag is detected across the coated region. These data support the presence of the intended components, but quantitative uniformity should be interpreted within the spatial resolution and detection limits of EDS.

The XPS survey spectrum contains signals assigned to *C* 1s, N 1s, O 1s, Ti 2p, and Ag 3d (Figure 4a). The reported *C* 1s components at 283.68 and 284.97 eV were assigned to *C*–*C* and *C*–O environments, respectively (Figure 4b). The N 1s components at 398.89 and 400.57 eV were assigned to N–H and N–*C* environments (Figure 4c). O 1s components at 529.75 and 531.56 eV were assigned to lattice Ti–O–Ti and surface/interfacial oxygen species, respectively (Figure 4d). Ti 2p peaks at 458.66 and 464.40 eV were assigned to Ti 2p_3/2_ and Ti 2p_1/2_ of Ti^4+^ (Figure 4e). Ag 3d peaks at 367.51 and 373.72 eV were assigned to Ag 3d_5/2_ and Ag 3d_3/2_ (Figure 4f). The Ag 3d spectrum was interpreted as consistent with predominantly metallic Ag. Metallic Ag may influence optical absorption and interfacial charge-transfer behavior in TiO_2_-based systems [45].

### 2.3. Optical Properties of THPP/TiO_2_/Ag@ACFag

Figure 5a compares the UV–vis diffuse-reflectance responses of TiO_2_ and THPP/TiO_2_/Ag@ACFag. The supported composite shows a higher response over a broader wavelength range than TiO_2_. This observation is consistent with additional absorption or scattering contributions from THPP, Ag, and ACF; the relative contributions of these components cannot be separated from the reported spectrum alone [46].

The optical-transition analysis was described using the general Tauc relation, calculated as Equation (1) [23]:

(1) $${\left(\alpha h\nu \right)}^{2}\;=\;A(h\nu \;-\;E_{g})$$

where *α* denotes the optical absorption coefficient, *hν* is the photon energy, *A* is a proportionality constant, *n* is the exponent selected for the assumed transition model, and *E_g_* denotes the fitted optical-transition energy.

The relationship between (*αhν*)^2^ and *hν* was illustrated in Figure 5b, which indicates intercepts of approximately 3.04 eV for TiO_2_ and 2.88 eV for THPP/TiO_2_/Ag@ACFag. The latter is described here as an apparent optical transition energy, not a uniquely determined intrinsic band gap of the multiphase composite.

Steady-state PL spectra were recorded to examine the optical emission behavior (Figure 5c). Obviously, the red-shift in the PL emission from 428.73 nm for TiO_2_ to 490.83 nm for THPP/TiO_2_/Ag@ACFag can be observed, which suggests that interfacial coupling among THPP, TiO_2_, Ag, and ACF modifies the excited-state relaxation pathways of the composite, possibly through the formation of additional interfacial or defect-related electronic states.

### 2.4. Photocatalytic Decolorization Performance and Proposed Interfacial Processes

Under irradiation, THPP and TiO_2_ absorb light and generate excited charge carriers, while the intimate interfaces among THPP, TiO_2_, Ag, and ACF may facilitate interfacial charge transfer and alter carrier recombination behavior [47,48]. Ag nanoparticles may act as electron-accepting or mediating sites, and the Ag/TiO_2_ interface may influence charge trapping and transfer through metal–semiconductor interactions [49]. Meanwhile, the conductive ACF framework may provide additional pathways for charge transport and also enrich MB through adsorption [50]. The photogenerated charge carriers can subsequently participate in surface redox reactions and generate reactive species that contribute to MB decolorization. Because radical-trapping and electron paramagnetic resonance measurements were not performed, the identities and relative contributions of individual reactive species cannot be specifically assigned. To evaluate the effect of the immersion cycles on photocatalytic performance, MB was used as a model pollutant, and its decolorization was monitored by UV–vis spectroscopy. Figure 6 and Table 1 and Table 2 compare the decolorization and apparent kinetic behavior of THPP/TiO_2_/Ag@ACFag under xenon-lamp and UV irradiation.

Figure 6a,c show a rapid initial decrease in the plotted concentration ratio followed by a slower decrease. The available records do not establish a unique cause for this change in slope. The one-immersion-cycle sample showed greater apparent removal at the end of each plotted experiment than the two-immersion-cycle sample. The kinetic behavior was analyzed using the apparent pseudo-first-order expression of Equation (2) [51]:

(2) $$\ln(C_{0}/C_{t})\;=\;kt$$

Here, *C*_0_ is the MB concentration after dark adsorption and immediately before irradiation, *C_t_* is the concentration after irradiation for time *t*, and k is the apparent pseudo-first-order rate constant.

Dark adsorption, irradiation-stage decolorization, and overall apparent removal were calculated using the distinct denominators defined in Section 2.4 and Equations (3)–(5) [52].

The concentration profiles were approximately fitted using an apparent pseudo-first-order model (Figure 6b,d). Under 300 W xenon-lamp irradiation, the reported k values were 0.00455 and 0.00240 min^−1^ for THPP/TiO_2_/Ag@ACFag-1 and THPP/TiO_2_/Ag@ACFag-2, with *R*^2^ values of 0.96993 and 0.98233, respectively. Under 6 W UV irradiation, the corresponding *k*_app_ values were 0.00343 and 0.00200 min^−1^, with *R*^2^ values of 0.83011 and 0.85931. The UV data therefore provide only an approximate kinetic trend, not confirmation of a kinetic law. In both reported fits, the one-immersion-cycle sample had the higher apparent rate constant; statistical significance was not established.

To compare the photocatalytic performance of THPP/TiO_2_/Ag@ACFag, representative TiO_2_, TiO_2_/carbon, porphyrin/TiO_2_, and Ag/TiO_2_ photocatalysts were compared using the apparent pseudo-first-order rate constant together with key experimental conditions, including light source, irradiance, pollutant concentration, catalyst dosage, solution volume, and irradiation time (Table 3) [38,53,54,55]. The present system is therefore evaluated by considering both its apparent kinetic behavior and its immobilized, adsorptive, and recoverable ACF-supported architecture. Overall, the comparison shows that the main advantage of the THPP/TiO_2_/Ag@ACFag system lies in integrated adsorption–photocatalysis function and immobilized, recoverable architecture.

In summary, the concentration changes of MB for both THPP/TiO_2_/Ag@ACFag-1 and THPP/TiO_2_/Ag@ACFag-2 under xenon-lamp and UV irradiation were fitted using an apparent pseudo-first-order kinetic model. Under both irradiation conditions, THPP/TiO_2_/Ag@ACFag-1 showed better photocatalytic decolorization performance than THPP/TiO_2_/Ag@ACFag-2, as reflected by its higher MB removal within the same irradiation period and larger apparent rate constant. From an application perspective, immobilization of the photocatalytic components on the ACF aerogel provides a macroscopic and recoverable material form, avoiding the need to separate fine photocatalyst powders from the treated solution. The ACF framework also contributes to MB adsorption, allowing pollutant enrichment and irradiation-induced decolorization to occur within the same material platform. These features make the composite relevant to reusable photocatalytic adsorbents and fixed photocatalytic units for water-treatment applications.

## 3. Conclusions

THPP/TiO_2_/Ag@ACF aerogel composites were successfully fabricated by hydrothermal synthesis followed by impregnation onto ACF aerogel. Structural characterization confirmed the incorporation of THPP, anatase TiO_2_, and metallic Ag, while UV–vis DRS and PL results demonstrated altered optical and excited-state behavior. The optimized THPP/TiO_2_/Ag@ACFag-1 sample achieved overall apparent MB removal efficiencies of 88.7% under 6 W UV irradiation and 88.0% under 300 W xenon-lamp irradiation, with a higher apparent pseudo-first-order rate constant than the two-cycle sample. The observed performance reflects the combined contributions of adsorption, photocatalytic decolorization, and the multiphase composite architecture. However, the present results do not support assignment to a specific Type-II, Schottky, or Z-scheme configuration. Overall, the ACF-supported composite provides a recoverable platform for photocatalytic pollutant removal.

## 4. Materials and Methods

### 4.1. Materials

THPP was obtained from Titan Scientific Co., Ltd. (Shanghai, China). Tetrahydrofuran was supplied by Zesheng Technology Co., Ltd. (Anqing, China); sodium dodecyl sulfate by Bide Pharmaceutical Technology Co., Ltd. (Shanghai, China); tetrabutyl titanate (TBT) by Zhanyun Chemical Co., Ltd. (Shanghai, China); and silver nanoparticles by Aladdin Biochemical Technology Co., Ltd. (Shanghai, China). Acetic acid, methylene blue (MB), and anhydrous ethanol were obtained from Xilong Scientific Co., Ltd. (Guangzhou, China). All reagents were used as received, and ultrapure water was used throughout the experiments.

### 4.2. Preparation of THPP/TiO_2_/Ag@ACFag

THPP powder (50 mg) and tetrabutyl titanate (250 μL) were dissolved in tetrahydrofuran (50 mL). Nano-Ag was added at a nominal 3 wt% relative to the total mass of the precursor formulation, and the mixture was stirred at 25 °C for 0.5 h. The solution was rapidly poured into 500 mL of an aqueous sodium dodecyl sulfate solution (0.01 M) under magnetic stirring and then stirred for 24 h. The mixture was centrifuged at 8000 rpm for 10 min to remove excess reactants. The precipitate was redispersed in water and transferred to a Teflon-lined autoclave for hydrothermal treatment at 180 °C for 16 h. This nominal synthesis composition does not represent the measured Ag content of the final supported composite. The resulting THPP/TiO_2_/Ag particles were collected by centrifugation at 8000 rpm for 10 min.

A 0.5% silane coupling-agent solution was prepared, and THPP/TiO_2_/Ag particles were dispersed in this solution. After ultrasonication for 30 min, ACF aerogel samples measuring 2 cm × 2 cm were immersed for one or two cycles and were marked as THPP/TiO_2_/Ag@ACFag-1 or THPP/TiO_2_/Ag@ACFag-2, respectively.

### 4.3. Characterization

Microstructure and elemental distribution were examined by field-emission scanning electron microscopy (SEM) (5 kV, GeminiSEM 300, Zeiss, Germany) coupled with energy-dispersive X-ray spectroscopy (EDS). Crystal structure was analyzed by X-ray diffraction (XRD, Cu Kα radiation, *λ* = 1.5406 Å; 2*θ* = 10–80°, Ultima IV, Rigaku, Akishima, Japan). Functional groups and surface chemical states were characterized by Fourier-transform infrared spectroscopy (FTIR, KBr pellet, 4000–600 cm^−1^, Nicolet is 10, Thermo Scientific, Waltham, MA, USA) and X-ray photoelectron spectroscopy (XPS, Al Kα source, binding energies calibrated to *C* 1s at 284.8 eV, Thermo Scientific, Waltham, MA, USA), respectively. Nanoscale structure was examined by high-resolution transmission electron microscopy (HRTEM, 200 kV, Talos F200X G2, Thermo Scientific, Waltham, MA, USA) with high-angle annular dark-field imaging. Optical properties were measured by UV–vis diffuse-reflectance spectroscopy (DRS, BaSO_4_ reference, 200–750 nm, UV-2600, Shimadzu, Kyoto, Japan), and photoluminescence (PL) spectra were collected using an spectrofluorometer (Cary Eclipse, Agilent, Santa Clara, CA, USA) at an excitation wavelength of 325 nm.

### 4.4. Photocatalytic Experiments

Photocatalytic experiments were conducted by immersing THPP/TiO_2_/Ag@ACFag (2 × 2 × 0.5 cm^3^) into MB solution (25 mL, 20 mg L^−1^), which was first kept in the dark for 90s to allow adsorption–desorption equilibration, then irradiated under a xenon lamp (CME SL500, China Micro Energy Technology Co., Ltd., Beijing, China) equipped with an AM 1.5G filter for 5 min during the photocatalytic experiments. The lamp system emission range was approximately 300–2500 nm, the irradiance at the sample position was approximately 100 mW cm^−2^, and the source-to-reactor distance was 15 cm. In addition, a 6 W UV lamp was also applied for supplementary experiments. During the whole period, the solution was stirred at 200 rpm.

To investigate MB decolorization, the concentration change under irradiation was monitored. Firstly, a standard curve of adsorption–concentration of MB solution was obtained through a predetermined series of MB solution concentrations. Afterwards, 3 mL of MB solution was collected at a predetermined irradiation time (1 min) and analyzed by UV–vis spectrophotometry to monitor MB solution concentration. Here, *C_i_* represents the initial MB concentration before dark adsorption, *C*_0_ represents the concentration after dark adsorption and immediately before irradiation, and *C_t_* represents the concentration after an irradiation time *t*. The irradiation-stage kinetics were evaluated using the apparent pseudo-first-order model ln(*C*_0_/*C_t_*) = *kt*, where *k* is the apparent pseudo-first-order rate constant [51]. Dark adsorption, irradiation-stage decolorization, and overall apparent removal were defined as follows [52]:

(3) $$\eta_{ads}\;=\;\frac{C_{i}\;-\;C_{0}}{C_{i}}\;\times \;100\%$$

(4) $$\eta_{photo}=\frac{C_{0}-C_{t}}{C_{0}}\times 100\%$$

(5) $$\eta_{overall}=\frac{C_{i}-C_{t}}{C_{i}}\times 100\%$$

## Figures and Tables

**Figure 2:**
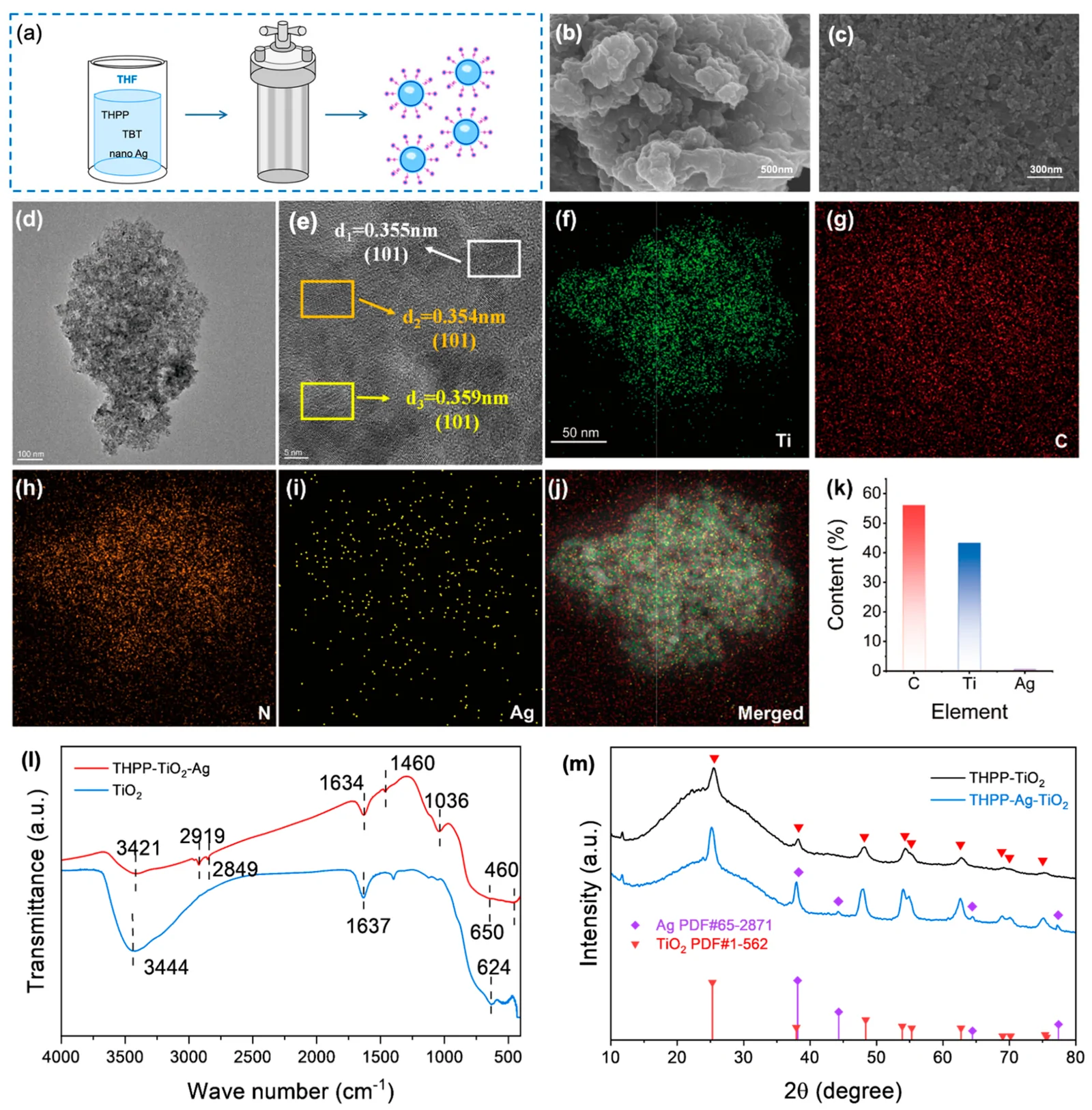
(**a**) Schematic preparation of THPP/TiO_2_/Ag powder. (**b**,**c**) SEM images of THPP/TiO_2_ and THPP/TiO_2_/Ag powders, respectively. (**d**) TEM and (**e**) HRTEM images of THPP/TiO_2_/Ag. (**f**–**i**) Ti, *C*, N, and Ag EDS elemental maps, respectively, and (**j**) merged map. (**k**) Elemental composition of THPP/TiO_2_/Ag. (**l**) FTIR spectra and (**m**) XRD patterns of THPP/TiO_2_/Ag and THPP/TiO_2_.

**Figure 3:**
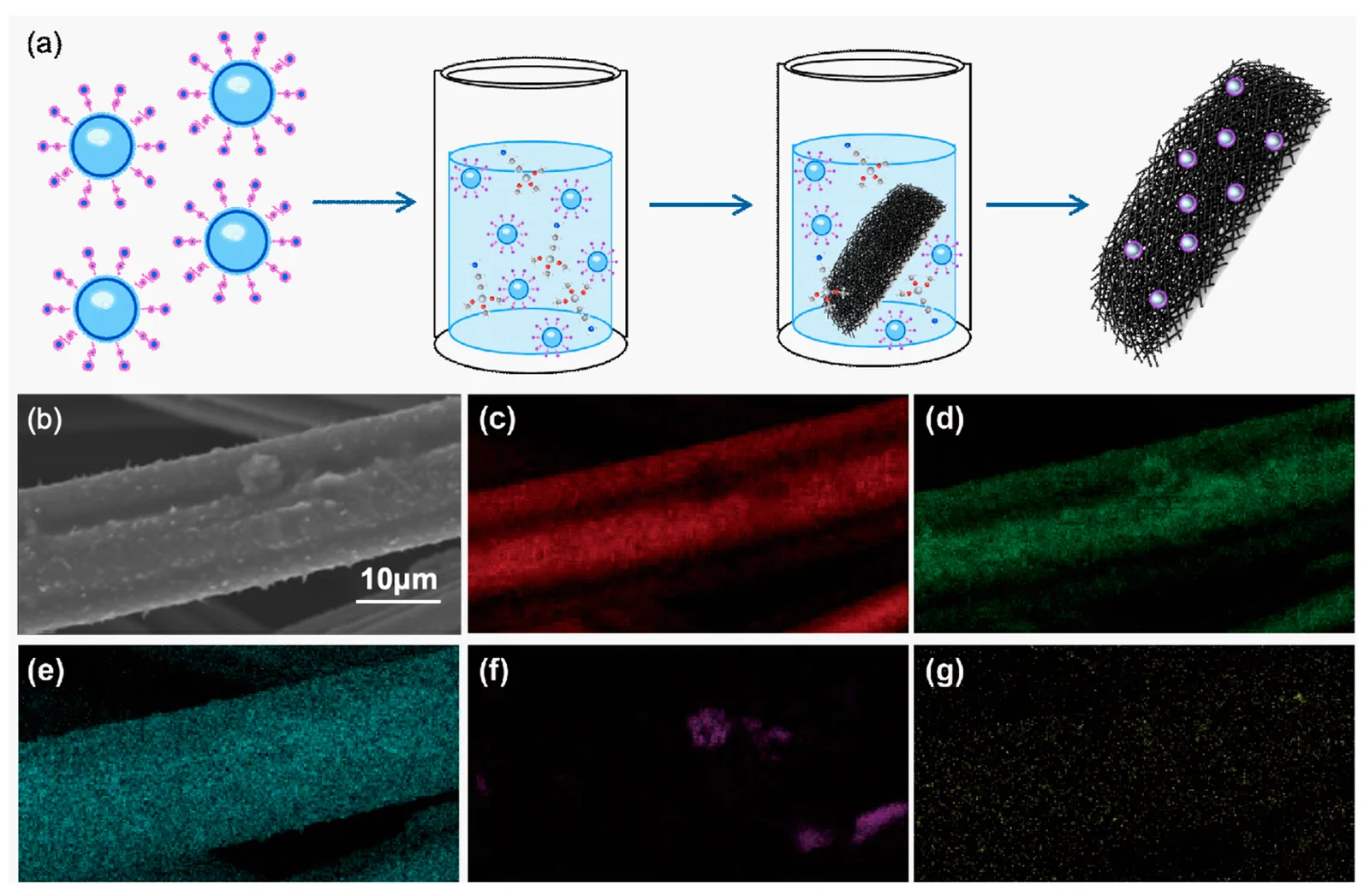
(**a**) Schematic preparation of THPP/TiO_2_/Ag@ACFag. (**b**) SEM image of the coated ACF. (**c**) *C*, (**d**) Ti, (**e**) O, (**f**) N, and (**g**) Ag EDS elemental maps of the THPP/TiO_2_/Ag@ACFag composite.

**Figure 4:**
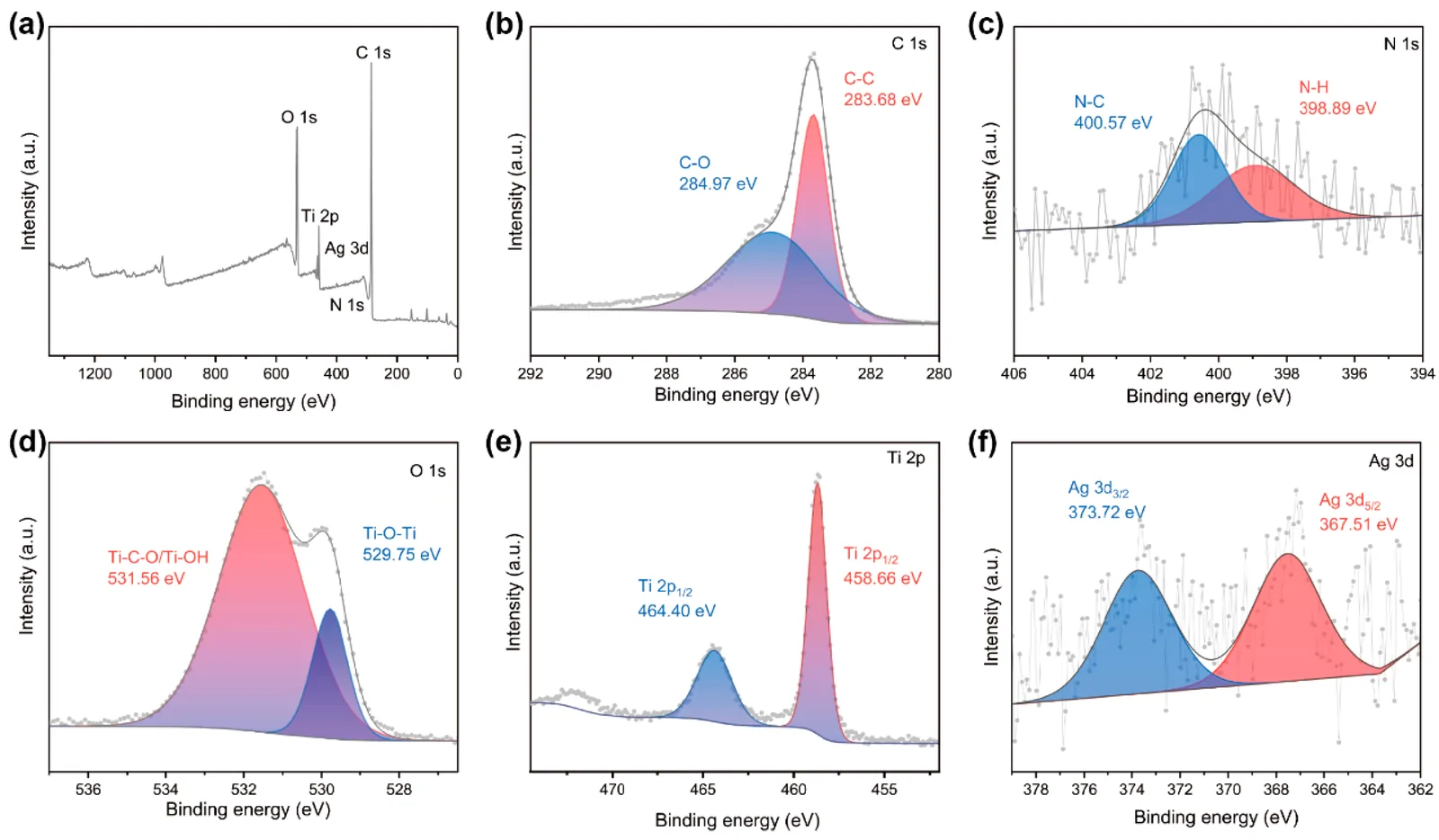
(**a**) XPS survey spectrum of THPP/TiO_2_/Ag@ACFag and high-resolution spectra of (**b**) C 1s, (**c**) N 1s, (**d**) O 1s, (**e**) Ti 2p, and (**f**) Ag 3d.

**Figure 5:**
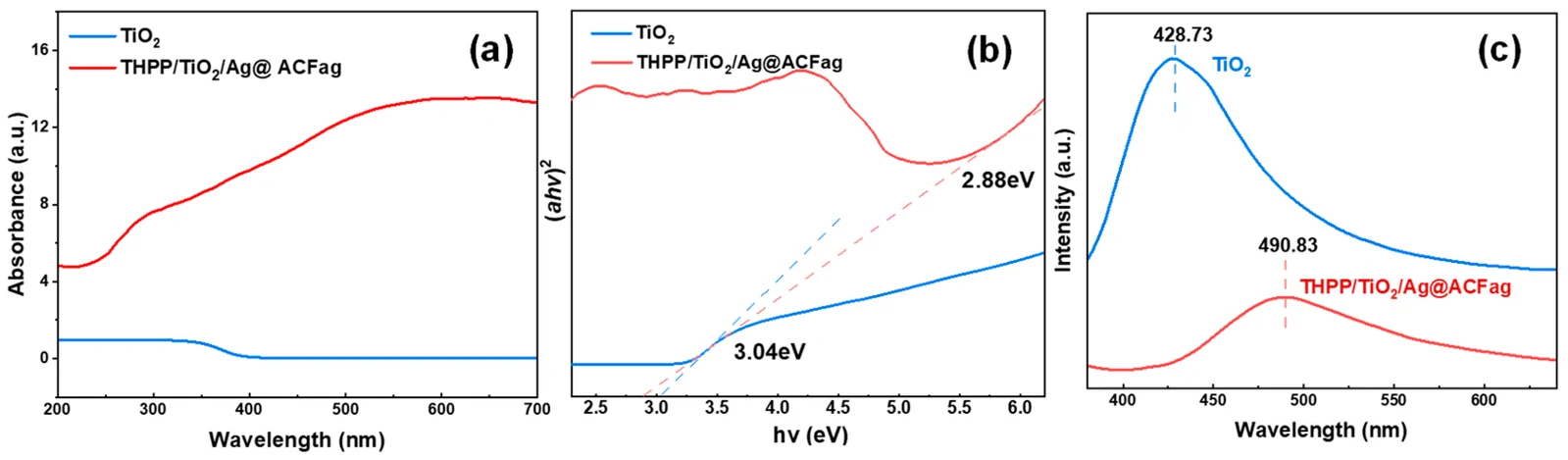
(**a**) UV–vis spectra of THPP/TiO_2_/Ag@ACFag and TiO_2_. (**b**) Relationship between (*αhν*)^2^ and *hν*. (**c**) Steady-state PL spectra of THPP/TiO_2_/Ag@ACFag and TiO_2_.

**Figure 6:**
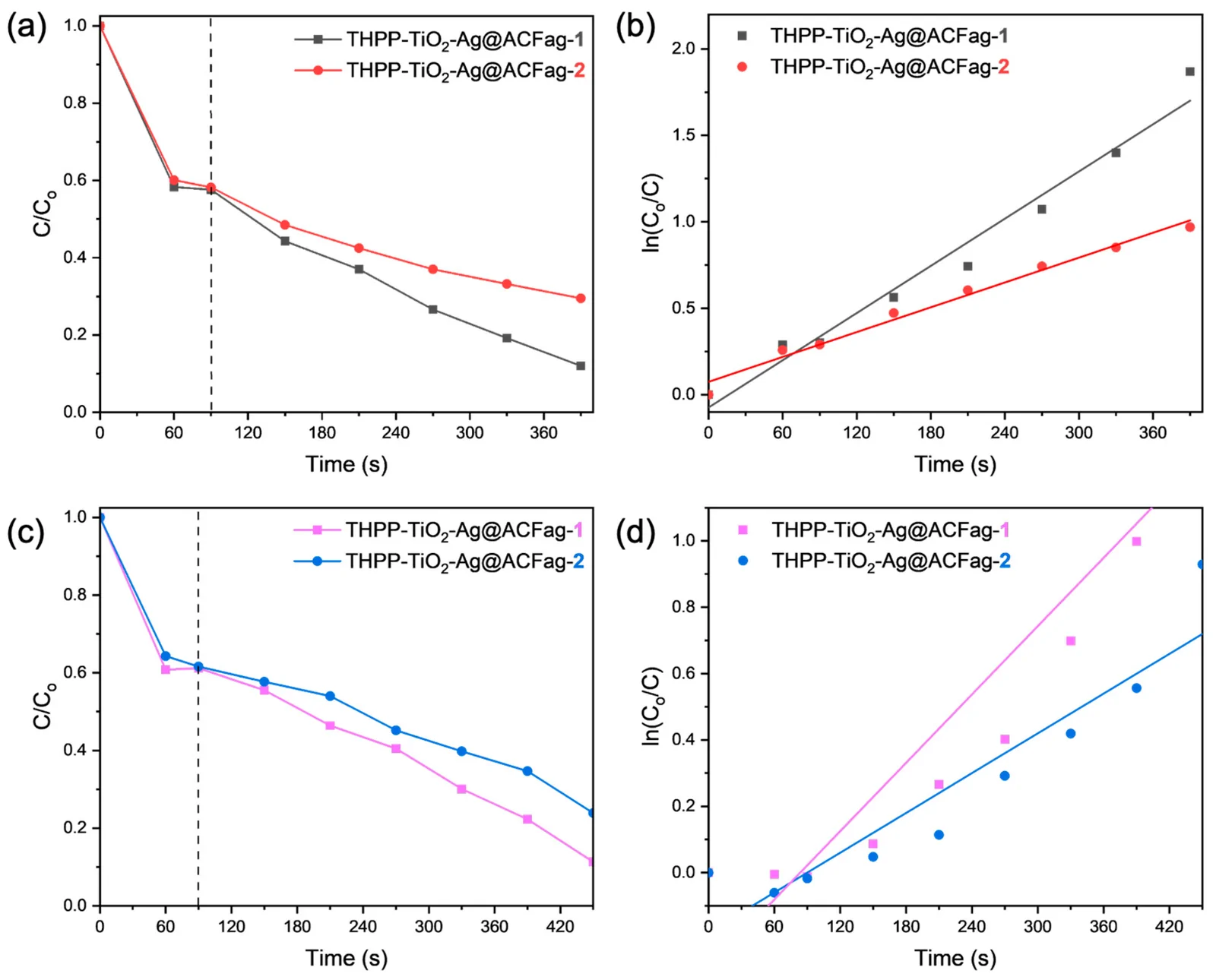
Photocatalytic performance of THPP/TiO_2_/Ag@ACFag composite. (**a**) Concentration profiles and (**b**) corresponding apparent pseudo-first-order plots under 300 W xenon-lamp irradiation. (**c**) Concentration profiles and (**d**) corresponding apparent pseudo-first-order plots under 6 W UV irradiation.

**Table 1:** Apparent pseudo-first-order rate constants and coefficients of determination for MB decolorization.

Sample	Light	k (min^−1^)	*R* ^2^
THPP/TiO_2_/Ag@ACFag-1	300 W xenon lamp	0.00455	0.96993
THPP/TiO_2_/Ag@ACFag-2	300 W xenon lamp	0.00240	0.98233
THPP/TiO_2_/Ag@ACFag-1	6 W UV lamp	0.00343	0.83011
THPP/TiO_2_/Ag@ACFag-2	6 W UV lamp	0.00200	0.85931

**Table 2:** Dark adsorption, irradiation-stage decolorization, and overall apparent MB removal by THPP/TiO_2_/Ag@ACFag composites for 5 min, calculated using Equations (3)–(5).

Sample	Light Condition	Dark Adsorption (%)	Irradiation-Stage Decolorization (%)	Overall Apparent Removal (%)
THPP/TiO_2_/Ag@ACFag-1	UV	38.8	63.6	77.7
THPP/TiO_2_/Ag@ACFag-1	xenon	42.4	79.2	88.0
THPP/TiO_2_/Ag@ACFag-2	UV	38.4	43.7	65.3
THPP/TiO_2_/Ag@ACFag-2	xenon	41.8	49.3	70.5

**Table 3:** Reported MB decolorization/removal and apparent kinetic constants for representative TiO_2_-based systems, with available experimental conditions. The porphyrin/TiO_2_ entry identifies an existing reference but has no verified MB comparison data in the supplied documents.

Material	Light/Spectrum	Irradiance	Initial MB	Catalyst Dosage	Solution Volume	Time	Apparent Removal/Decolorization	*k* (min^−1^)	Ref.
TiO_2_	Solar simulator,1 sun	NR	2 mg L^−1^	100 ppm	NR	50 min	≈26.7%	0.0062	[53]
Ag/TiO_2_	Solar simulator,1 sun	NR	2 mg L^−1^	100 ppm	NR	50 min	≈52.5%	0.0149	[53]
Fe-TiO_2_/biochar	visible light	NR	10 mg L^−1^	1.0 g L^−1^	NR	180 min	83%	0.0103	[54]
TiO_2_/RGO	Natural solar	NR	30 mg L^−1^	1.0 g L^−1^	NR	138 min	97.0%	0.0151	[55]
Porphyrin/TiO_2_	NR	NR	NR	NR	NR	NR	NR	NR	[38]
THPP/TiO_2_/Ag@ACFag-1	300 W xenon lamp	≈100 mW cm^−2^	20 mg L^−1^	One immobilized composite (2 × 2 × 0.5 cm^3^)	NR	5 min	88.0%	0.00455	This work
THPP/TiO_2_/Ag@ACFag-1	6 W UV lamp	NR	20 mg L^−1^	One immobilized composite (2 × 2 × 0.5 cm^3^)	NR	6 min	88.7%	0.00343	This work

## Data Availability

Data are available from the corresponding authors upon reasonable request.
